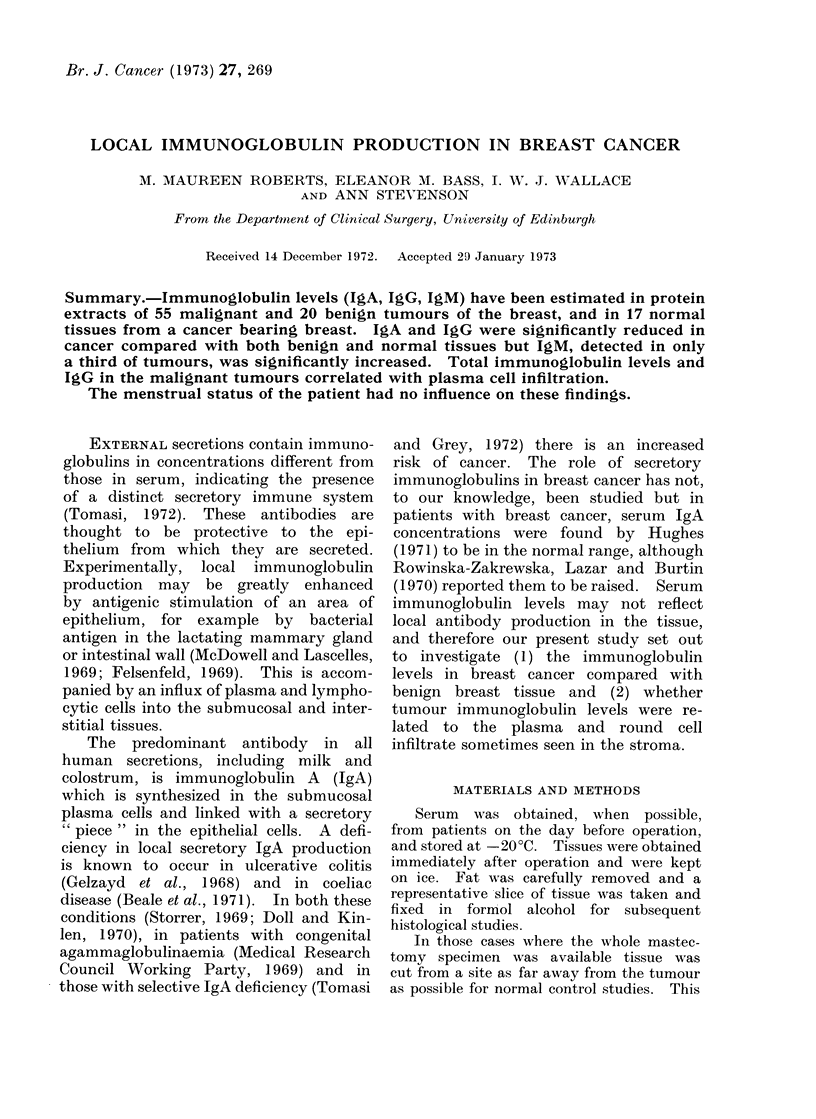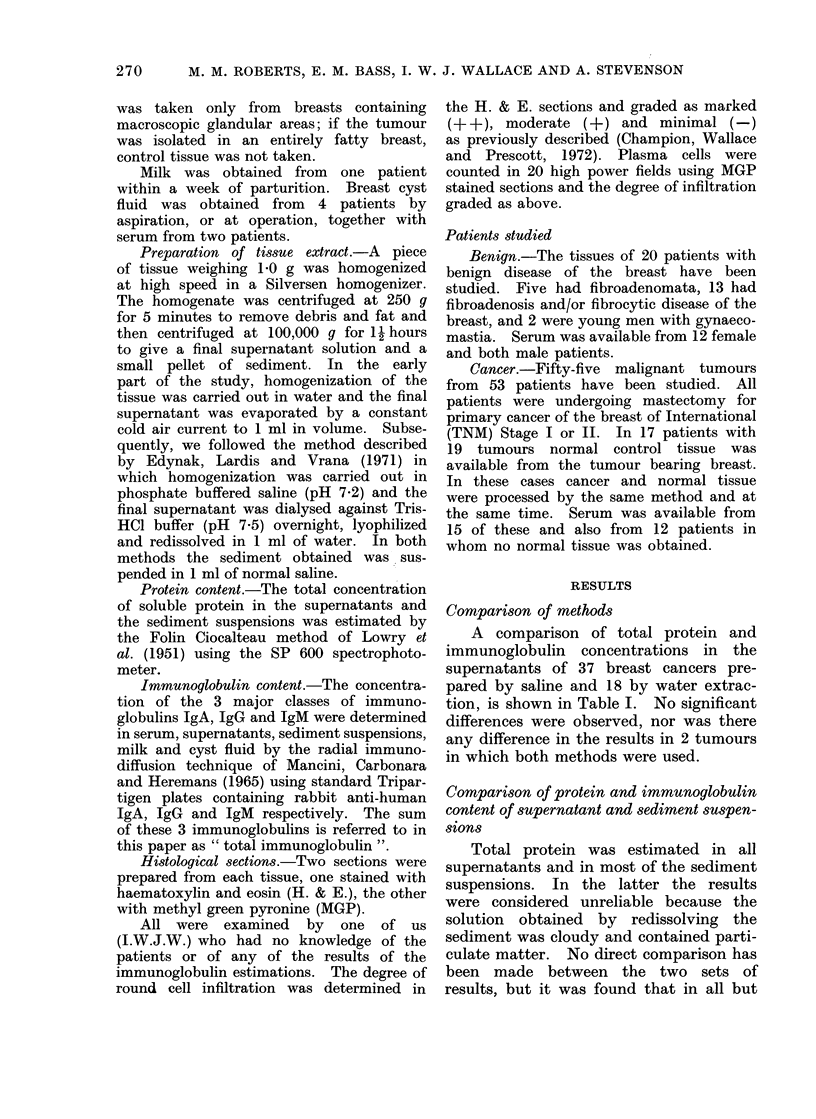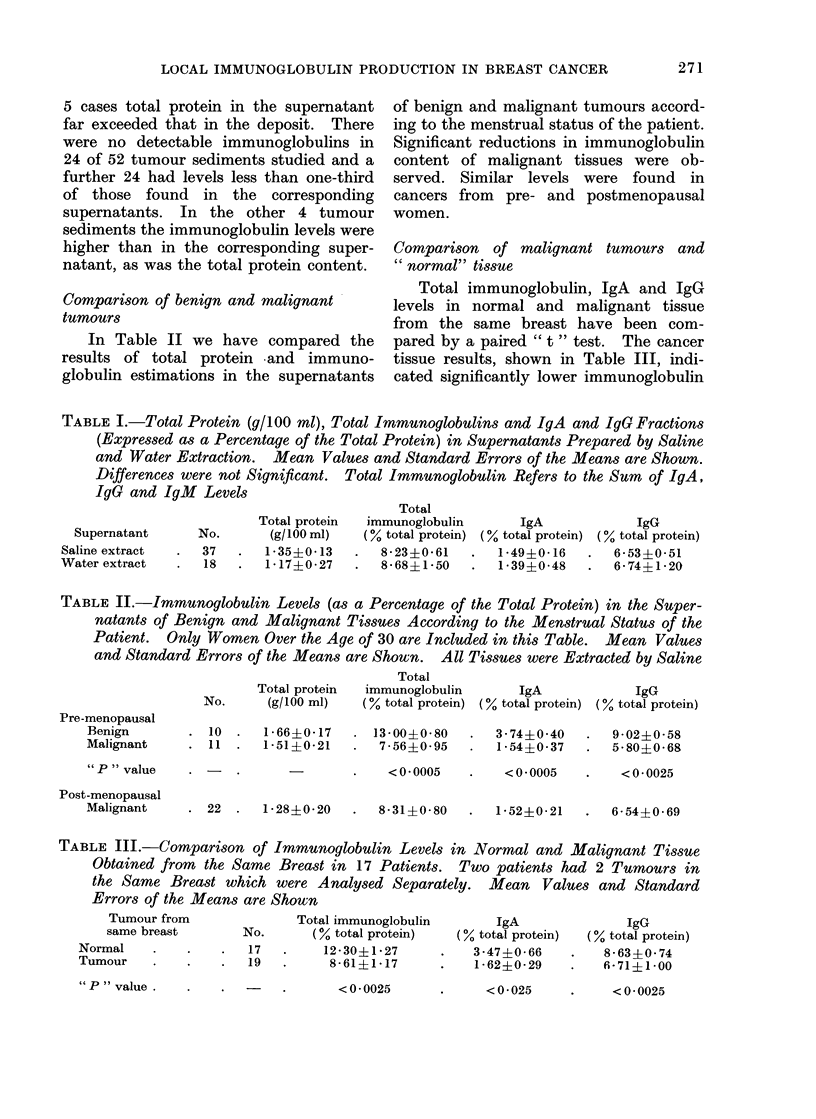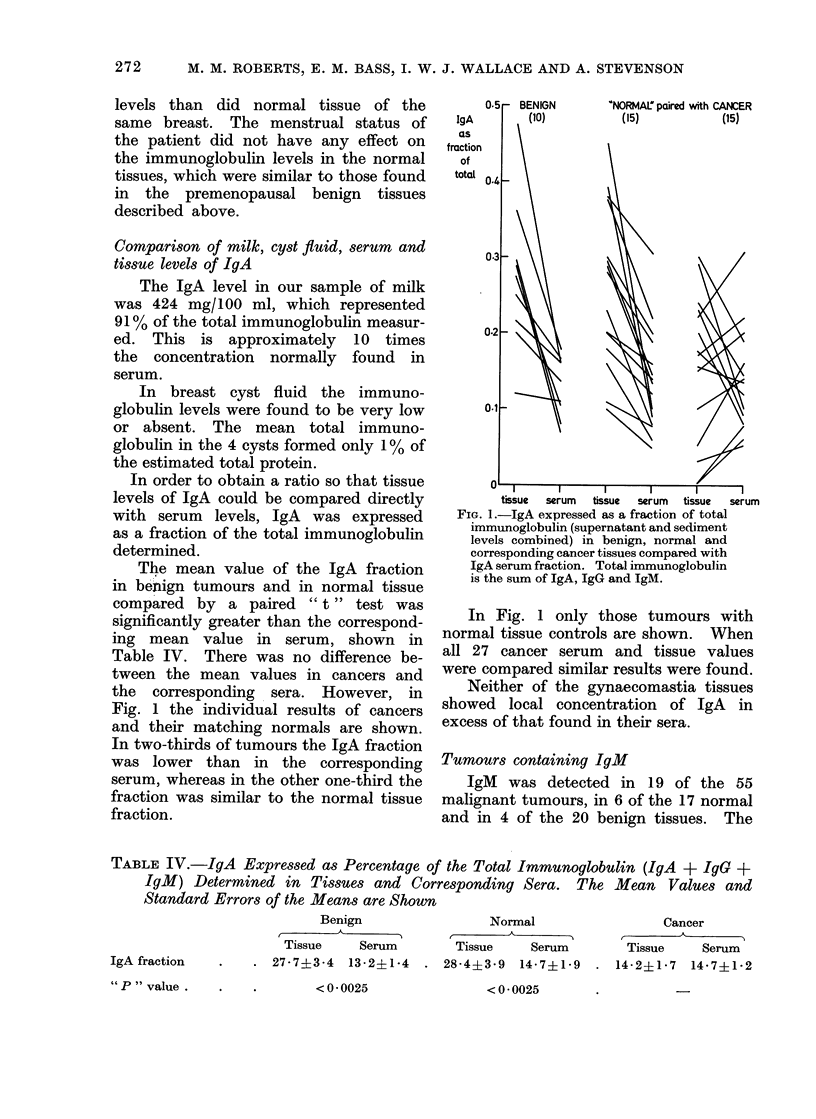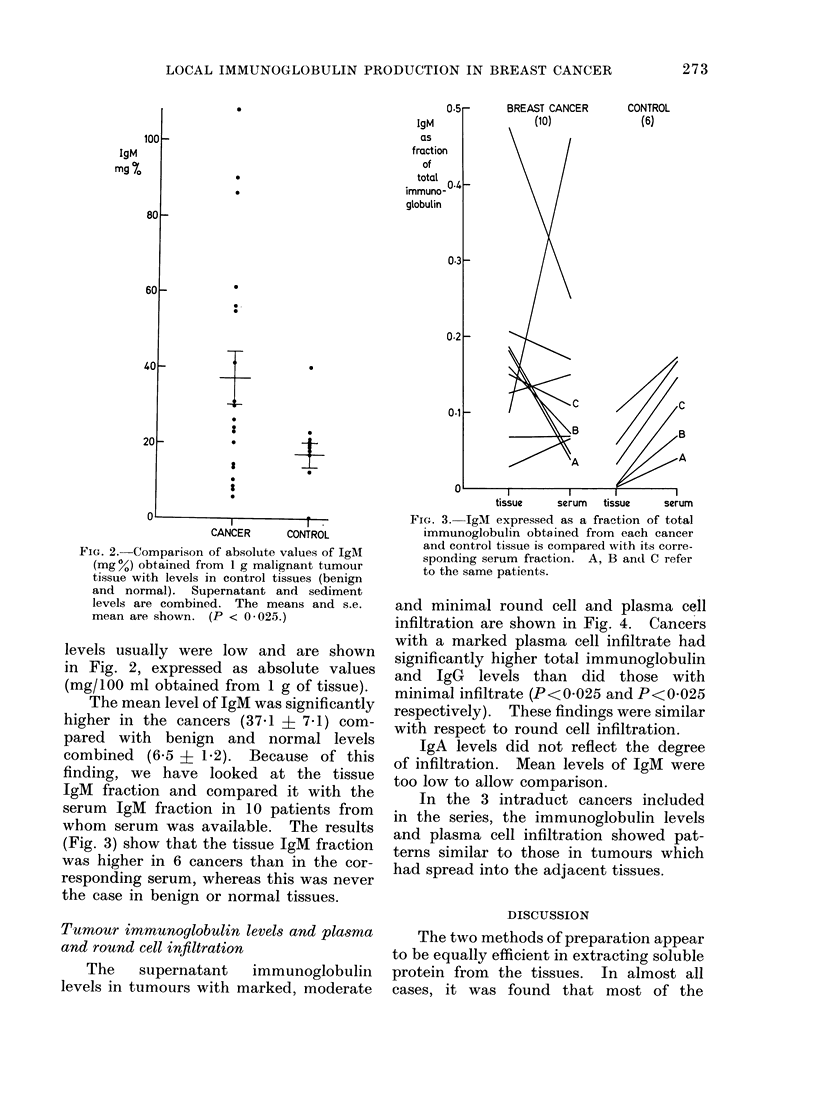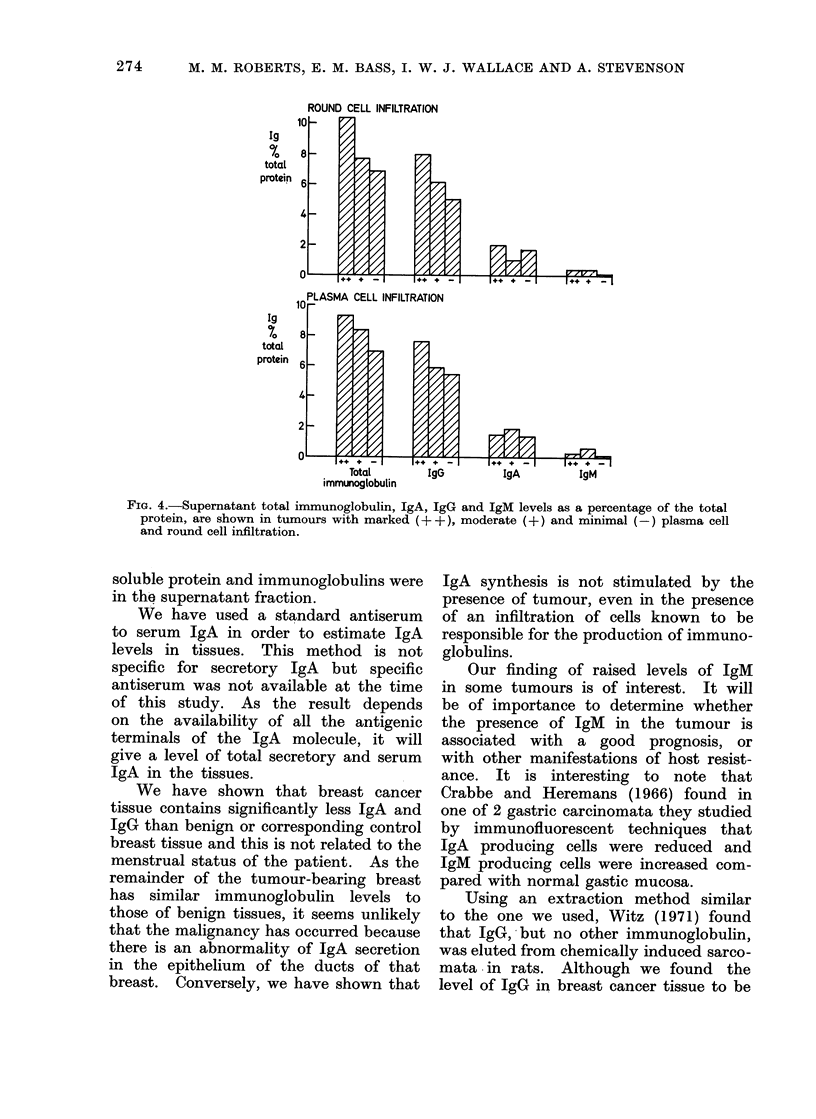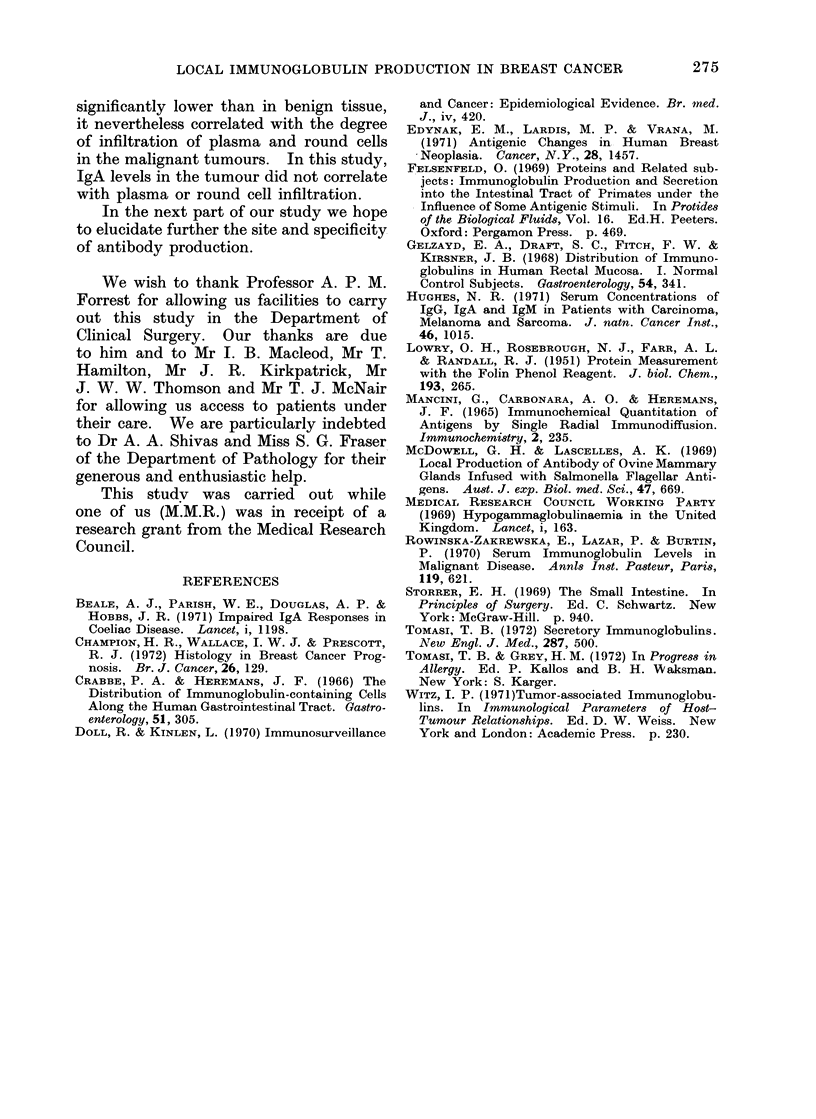# Local Immunoglobulin Production in Breast Cancer

**DOI:** 10.1038/bjc.1973.33

**Published:** 1973-04

**Authors:** M. Maureen Roberts, Eleanor M. Bass, I. W. J. Wallace, Ann Stevenson

## Abstract

Immunoglobulin levels (IgA, IgG, IgM) have been estimated in protein extracts of 55 malignant and 20 benign tumours of the breast, and in 17 normal tissues from a cancer bearing breast. IgA and IgG were significantly reduced in cancer compared with both benign and normal tissues but IgM, detected in only a third of tumours, was significantly increased. Total immunoglobulin levels and IgG in the malignant tumours correlated with plasma cell infiltration.

The menstrual status of the patient had no influence on these findings.


Br. J. Cancer (1973) 27, 269

### LOCAL IMMUNOGLOBULIN PRODUCTION IN BREAST CANCER

M. MAUREEN ROBERTS, ELEANOR M. BASS, I. W. J. WALLACE AND ANN STEVENSON

*From the Department of Clinical Surgery, University of Edinburgh*

Received 14 December 1972. Accepted 29 January 1973

**Summary.—Immunoglobulin levels (IgA, IgG, IgM) have been estimated in protein extracts of 55 malignant and 20 benign tumours of the breast, and in 17 normal tissues from a cancer bearing breast. IgA and IgG were significantly reduced in cancer compared with both benign and normal tissues but IgM, detected in only a third of tumours, was significantly increased. Total immunoglobulin levels and IgG in the malignant tumours correlated with plasma cell infiltration.**

**The menstrual status of the patient had no influence on these findings.**

EXTERNAL secretions contain immunoglobulins in concentrations different from those in serum, indicating the presence of a distinct secretory immune system (Tomasi, 1972). These antibodies are thought to be protective to the epithelium from which they are secreted. Experimentally, local immunoglobulin production may be greatly enhanced by antigenic stimulation of an area of epithelium, for example by bacterial antigen in the lactating mammary gland or intestinal wall (McDowell and Lascelles, 1969; Felsenfeld, 1969). This is accompanied by an influx of plasma and lymphocytic cells into the submucosal and interstitial tissues.

The predominant antibody in all human secretions, including milk and colostrum, is immunoglobulin A (IgA) which is synthesized in the submucosal plasma cells and linked with a secretory " piece " in the epithelial cells. A deficiency in local secretory IgA production is known to occur in ulcerative colitis (Gelzayd *et al.*, 1968) and in coeliac disease (Beale *et al.*, 1971). In both these conditions (Storrer, 1969; Doll and Kinlen, 1970), in patients with congenital agammaglobulinaemia (Medical Research Council Working Party, 1969) and in those with selective IgA deficiency (Tomasi and Grey, 1972) there is an increased risk of cancer. The role of secretory immunoglobulins in breast cancer has not, to our knowledge, been studied but in patients with breast cancer, serum IgA concentrations were found by Hughes (1971) to be in the normal range, although Rowinska-Zakrewska, Lazar and Burtin (1970) reported them to be raised. Serum immunoglobulin levels may not reflect local antibody production in the tissue, and therefore our present study set out to investigate (1) the immunoglobulin levels in breast cancer compared with benign breast tissue and (2) whether tumour immunoglobulin levels were related to the plasma and round cell infiltrate sometimes seen in the stroma.

### MATERIALS AND METHODS

Serum was obtained, when possible, from patients on the day before operation, and stored at −20°C. Tissues were obtained immediately after operation and were kept on ice. Fat was carefully removed and a representative slice of tissue was taken and fixed in formol alcohol for subsequent histological studies.

In those cases where the whole mastectomy specimen was available tissue was cut from a site as far away from the tumour as possible for normal control studies. This was taken only from breasts containing macroscopic glandular areas; if the tumour was isolated in an entirely fatty breast, control tissue was not taken.

Milk was obtained from one patient within a week of parturition. Breast cyst fluid was obtained from 4 patients by aspiration, or at operation, together with serum from two patients.

*Preparation of tissue extract.*—A piece of tissue weighing 1·0 g was homogenized at high speed in a Silversen homogenizer. The homogenate was centrifuged at 250 *g* for 5 minutes to remove debris and fat and then centrifuged at 100,000 *g* for 1½ hours to give a final supernatant solution and a small pellet of sediment. In the early part of the study, homogenization of the tissue was carried out in water and the final supernatant was evaporated by a constant cold air current to 1 ml in volume. Subsequently, we followed the method described by Edynak, Lardis and Vrana (1971) in which homogenization was carried out in phosphate buffered saline (pH 7·2) and the final supernatant was dialysed against Tris-HCl buffer (pH 7·5) overnight, lyophilized and redissolved in 1 ml of water. In both methods the sediment obtained was suspended in 1 ml of normal saline.

*Protein content.*—The total concentration of soluble protein in the supernatants and the sediment suspensions was estimated by the Folin Ciocalteau method of Lowry *et al.* (1951) using the SP 600 spectrophotometer.

*Immunoglobulin content.*—The concentration of the 3 major classes of immunoglobulins IgA, IgG and IgM were determined in serum, supernatants, sediment suspensions, milk and cyst fluid by the radial immunodiffusion technique of Mancini, Carbonara and Heremans (1965) using standard Tripartigen plates containing rabbit anti-human IgA, IgG and IgM respectively. The sum of these 3 immunoglobulins is referred to in this paper as " total immunoglobulin ".

*Histological sections.*—Two sections were prepared from each tissue, one stained with haematoxylin and eosin (H. & E.), the other with methyl green pyronine (MGP).

All were examined by one of us (I.W.J.W.) who had no knowledge of the patients or of any of the results of the immunoglobulin estimations. The degree of round cell infiltration was determined in the H. & E. sections and graded as marked (++), moderate (+) and minimal (−) as previously described (Champion, Wallace and Prescott, 1972). Plasma cells were counted in 20 high power fields using MGP stained sections and the degree of infiltration graded as above.

### *Patients studied*

*Benign.*—The tissues of 20 patients with benign disease of the breast have been studied. Five had fibroadenomata, 13 had fibroadenosis and/or fibrocytic disease of the breast, and 2 were young men with gynaecomastia. Serum was available from 12 female and both male patients.

*Cancer.*—Fifty-five malignant tumours from 53 patients have been studied. All patients were undergoing mastectomy for primary cancer of the breast of International (TNM) Stage I or II. In 17 patients with 19 tumours normal control tissue was available from the tumour bearing breast. In these cases cancer and normal tissue were processed by the same method and at the same time. Serum was available from 15 of these and also from 12 patients in whom no normal tissue was obtained.

### RESULTS

### *Comparison of methods*

A comparison of total protein and immunoglobulin concentrations in the supernatants of 37 breast cancers prepared by saline and 18 by water extraction, is shown in Table I. No significant differences were observed, nor was there any difference in the results in 2 tumours in which both methods were used.

### *Comparison of protein and immunoglobulin content of supernatant and sediment suspensions*

Total protein was estimated in all supernatants and in most of the sediment suspensions. In the latter the results were considered unreliable because the solution obtained by redissolving the sediment was cloudy and contained particulate matter. No direct comparison has been made between the two sets of results, but it was found that in all but

5 cases total protein in the supernatant far exceeded that in the deposit. There were no detectable immunoglobulins in 24 of 52 tumour sediments studied and a further 24 had levels less than one-third of those found in the corresponding supernatants. In the other 4 tumour sediments the immunoglobulin levels were higher than in the corresponding supernatant, as was the total protein content.

*Comparison of benign and malignant tumours*

In Table II we have compared the results of total protein and immunoglobulin estimations in the supernatants of benign and malignant tumours according to the menstrual status of the patient. Significant reductions in immunoglobulin content of malignant tissues were observed. Similar levels were found in cancers from pre- and postmenopausal women.

*Comparison of malignant tumours and "normal" tissue*

Total immunoglobulin, IgA and IgG levels in normal and malignant tissue from the same breast have been compared by a paired "t" test. The cancer tissue results, shown in Table III, indicated significantly lower immunoglobulin

TABLE I.—*Total Protein (g/100 ml), Total Immunoglobulins and IgA and IgG Fractions (Expressed as a Percentage of the Total Protein) in Supernatants Prepared by Saline and Water Extraction. Mean Values and Standard Errors of the Means are Shown. Differences were not Significant. Total Immunoglobulin Refers to the Sum of IgA, IgG and IgM Levels*

| Supernatant | No. | Total protein (g/100 ml) | Total immunoglobulin (% total protein) | IgA (% total protein) | IgG (% total protein) |
|---|---|---|---|---|---|
| Saline extract | 37 | 1·35±0·13 | 8·23±0·61 | 1·49±0·16 | 6·53±0·51 |
| Water extract | 18 | 1·17±0·27 | 8·68±1·50 | 1·39±0·48 | 6·74±1·20 |

TABLE II.—*Immunoglobulin Levels (as a Percentage of the Total Protein) in the Supernatants of Benign and Malignant Tissues According to the Menstrual Status of the Patient. Only Women Over the Age of 30 are Included in this Table. Mean Values and Standard Errors of the Means are Shown. All Tissues were Extracted by Saline*

| | No. | Total protein (g/100 ml) | Total immunoglobulin (% total protein) | IgA (% total protein) | IgG (% total protein) |
|---|---|---|---|---|---|
| Pre-menopausal | | | | | |
| Benign | 10 | 1·66±0·17 | 13·00±0·80 | 3·74±0·40 | 9·02±0·58 |
| Malignant | 11 | 1·51±0·21 | 7·56±0·95 | 1·54±0·37 | 5·80±0·68 |
| "*P*" value | — | — | <0·0005 | <0·0005 | <0·0025 |
| Post-menopausal | | | | | |
| Malignant | 22 | 1·28±0·20 | 8·31±0·80 | 1·52±0·21 | 6·54±0·69 |

TABLE III.—*Comparison of Immunoglobulin Levels in Normal and Malignant Tissue Obtained from the Same Breast in 17 Patients. Two patients had 2 Tumours in the Same Breast which were Analysed Separately. Mean Values and Standard Errors of the Means are Shown*

| Tumour from same breast | No. | Total immunoglobulin (% total protein) | IgA (% total protein) | IgG (% total protein) |
|---|---|---|---|---|
| Normal | 17 | 12·30±1·27 | 3·47±0·66 | 8·63±0·74 |
| Tumour | 19 | 8·61±1·17 | 1·62±0·29 | 6·71±1·00 |
| "*P*" value | — | <0·0025 | <0·025 | <0·0025 |

levels than did normal tissue of the same breast. The menstrual status of the patient did not have any effect on the immunoglobulin levels in the normal tissues, which were similar to those found in the premenopausal benign tissues described above.

*Comparison of milk, cyst fluid, serum and tissue levels of IgA*

The IgA level in our sample of milk was 424 mg/100 ml, which represented 91% of the total immunoglobulin measured. This is approximately 10 times the concentration normally found in serum.

In breast cyst fluid the immunoglobulin levels were found to be very low or absent. The mean total immunoglobulin in the 4 cysts formed only 1% of the estimated total protein.

In order to obtain a ratio so that tissue levels of IgA could be compared directly with serum levels, IgA was expressed as a fraction of the total immunoglobulin determined.

The mean value of the IgA fraction in benign tumours and in normal tissue compared by a paired "t" test was significantly greater than the corresponding mean value in serum, shown in Table IV. There was no difference between the mean values in cancers and the corresponding sera. However, in Fig. 1 the individual results of cancers and their matching normals are shown. In two-thirds of tumours the IgA fraction was lower than in the corresponding serum, whereas in the other one-third the fraction was similar to the normal tissue fraction.

FIG. 1.—IgA expressed as a fraction of total immunoglobulin (supernatant and sediment levels combined) in benign, normal and corresponding cancer tissues compared with IgA serum fraction. Total immunoglobulin is the sum of IgA, IgG and IgM.

In Fig. 1 only those tumours with normal tissue controls are shown. When all 27 cancer serum and tissue values were compared similar results were found.

Neither of the gynaecomastia tissues showed local concentration of IgA in excess of that found in their sera.

*Tumours containing IgM*

IgM was detected in 19 of the 55 malignant tumours, in 6 of the 17 normal and in 4 of the 20 benign tissues. The

TABLE IV.—*IgA Expressed as Percentage of the Total Immunoglobulin (IgA + IgG + IgM) Determined in Tissues and Corresponding Sera. The Mean Values and Standard Errors of the Means are Shown*

| | Benign | | Normal | | Cancer | |
|---|---|---|---|---|---|---|
| | Tissue | Serum | Tissue | Serum | Tissue | Serum |
| IgA fraction | 27·7±3·4 | 13·2±1·4 | 28·4±3·9 | 14·7±1·9 | 14·2±1·7 | 14·7±1·2 |
| "P" value | <0·0025 | | <0·0025 | | — | |

FIG. 2.—Comparison of absolute values of IgM (mg %) obtained from 1 g malignant tumour tissue with levels in control tissues (benign and normal). Supernatant and sediment levels are combined. The means and s.e. mean are shown. ($P < 0 \cdot 025$.)

FIG. 3.—IgM expressed as a fraction of total immunoglobulin obtained from each cancer and control tissue is compared with its corresponding serum fraction. A, B and C refer to the same patients.

levels usually were low and are shown in Fig. 2, expressed as absolute values (mg/100 ml obtained from 1 g of tissue).

The mean level of IgM was significantly higher in the cancers ($37 \cdot 1 \pm 7 \cdot 1$) compared with benign and normal levels combined ($6 \cdot 5 \pm 1 \cdot 2$). Because of this finding, we have looked at the tissue IgM fraction and compared it with the serum IgM fraction in 10 patients from whom serum was available. The results (Fig. 3) show that the tissue IgM fraction was higher in 6 cancers than in the corresponding serum, whereas this was never the case in benign or normal tissues.

*Tumour immunoglobulin levels and plasma and round cell infiltration*

The supernatant immunoglobulin levels in tumours with marked, moderate and minimal round cell and plasma cell infiltration are shown in Fig. 4. Cancers with a marked plasma cell infiltrate had significantly higher total immunoglobulin and IgG levels than did those with minimal infiltrate ($P<0 \cdot 025$ and $P<0 \cdot 025$ respectively). These findings were similar with respect to round cell infiltration.

IgA levels did not reflect the degree of infiltration. Mean levels of IgM were too low to allow comparison.

In the 3 intraduct cancers included in the series, the immunoglobulin levels and plasma cell infiltration showed patterns similar to those in tumours which had spread into the adjacent tissues.

### DISCUSSION

The two methods of preparation appear to be equally efficient in extracting soluble protein from the tissues. In almost all cases, it was found that most of the

FIG. 4.—Supernatant total immunoglobulin, IgA, IgG and IgM levels as a percentage of the total protein, are shown in tumours with marked (++), moderate (+) and minimal (−) plasma cell and round cell infiltration.

soluble protein and immunoglobulins were in the supernatant fraction.

We have used a standard antiserum to serum IgA in order to estimate IgA levels in tissues. This method is not specific for secretory IgA but specific antiserum was not available at the time of this study. As the result depends on the availability of all the antigenic terminals of the IgA molecule, it will give a level of total secretory and serum IgA in the tissues.

We have shown that breast cancer tissue contains significantly less IgA and IgG than benign or corresponding control breast tissue and this is not related to the menstrual status of the patient. As the remainder of the tumour-bearing breast has similar immunoglobulin levels to those of benign tissues, it seems unlikely that the malignancy has occurred because there is an abnormality of IgA secretion in the epithelium of the ducts of that breast. Conversely, we have shown that IgA synthesis is not stimulated by the presence of tumour, even in the presence of an infiltration of cells known to be responsible for the production of immunoglobulins.

Our finding of raised levels of IgM in some tumours is of interest. It will be of importance to determine whether the presence of IgM in the tumour is associated with a good prognosis, or with other manifestations of host resistance. It is interesting to note that Crabbe and Heremans (1966) found in one of 2 gastric carcinomata they studied by immunofluorescent techniques that IgA producing cells were reduced and IgM producing cells were increased compared with normal gastic mucosa.

Using an extraction method similar to the one we used, Witz (1971) found that IgG, but no other immunoglobulin, was eluted from chemically induced sarcomata in rats. Although we found the level of IgG in breast cancer tissue to be significantly lower than in benign tissue, it nevertheless correlated with the degree of infiltration of plasma and round cells in the malignant tumours. In this study, IgA levels in the tumour did not correlate with plasma or round cell infiltration.

In the next part of our study we hope to elucidate further the site and specificity of antibody production.

We wish to thank Professor A. P. M. Forrest for allowing us facilities to carry out this study in the Department of Clinical Surgery. Our thanks are due to him and to Mr I. B. Macleod, Mr T. Hamilton, Mr J. R. Kirkpatrick, Mr J. W. W. Thomson and Mr T. J. McNair for allowing us access to patients under their care. We are particularly indebted to Dr A. A. Shivas and Miss S. G. Fraser of the Department of Pathology for their generous and enthusiastic help.

This study was carried out while one of us (M.M.R.) was in receipt of a research grant from the Medical Research Council.

### REFERENCES

BEALE, A. J., PARISH, W. E., DOUGLAS, A. P. & HOBBS, J. R. (1971) Impaired IgA Responses in Coeliac Disease. *Lancet*, i, 1198.

CHAMPION, H. R., WALLACE, I. W. J. & PRESCOTT, R. J. (1972) Histology in Breast Cancer Prognosis. *Br. J. Cancer*, **26**, 129.

CRABBE, P. A. & HEREMANS, J. F. (1966) The Distribution of Immunoglobulin-containing Cells Along the Human Gastrointestinal Tract. *Gastroenterology*, **51**, 305.

DOLL, R. & KINLEN, L. (1970) Immunosurveillance and Cancer: Epidemiological Evidence. *Br. med. J.*, iv, 420.

EDYNAK, E. M., LARDIS, M. P. & VRANA, M. (1971) Antigenic Changes in Human Breast Neoplasia. *Cancer, N.Y.*, **28**, 1457.

FELSENFELD, O. (1969) Proteins and Related subjects: Immunoglobulin Production and Secretion into the Intestinal Tract of Primates under the Influence of Some Antigenic Stimuli. In *Protides of the Biological Fluids*, Vol. 16. Ed.H. Peeters. Oxford: Pergamon Press. p. 469.

GELZAYD, E. A., DRAFT, S. C., FITCH, F. W. & KIRSNER, J. B. (1968) Distribution of Immunoglobulins in Human Rectal Mucosa. I. Normal Control Subjects. *Gastroenterology*, **54**, 341.

HUGHES, N. R. (1971) Serum Concentrations of IgG, IgA and IgM in Patients with Carcinoma, Melanoma and Sarcoma. *J. natn. Cancer Inst.*, **46**, 1015.

LOWRY, O. H., ROSEBROUGH, N. J., FARR, A. L. & RANDALL, R. J. (1951) Protein Measurement with the Folin Phenol Reagent. *J. biol. Chem.*, **193**, 265.

MANCINI, G., CARBONARA, A. O. & HEREMANS, J. F. (1965) Immunochemical Quantitation of Antigens by Single Radial Immunodiffusion. *Immunochemistry*, **2**, 235.

MCDOWELL, G. H. & LASCELLES, A. K. (1969) Local Production of Antibody of Ovine Mammary Glands Infused with Salmonella Flagellar Antigens. *Aust. J. exp. Biol. med. Sci.*, **47**, 669.

MEDICAL RESEARCH COUNCIL WORKING PARTY (1969) Hypogammaglobulinaemia in the United Kingdom. *Lancet*, i, 163.

ROWINSKA-ZAKREWSKA, E., LAZAR, P. & BURTIN, P. (1970) Serum Immunoglobulin Levels in Malignant Disease. *Annls Inst. Pasteur, Paris*, **119**, 621.

STORRER, E. H. (1969) The Small Intestine. In *Principles of Surgery*. Ed. C. Schwartz. New York: McGraw-Hill. p. 940.

TOMASI, T. B. (1972) Secretory Immunoglobulins. *New Engl. J. Med.*, **287**, 500.

TOMASI, T. B. & GREY, H. M. (1972) In *Progress in Allergy*. Ed. P. Kallos and B. H. Waksman. New York: S. Karger.

WITZ, I. P. (1971)Tumor-associated Immunoglobulins. In *Immunological Parameters of Host–Tumour Relationships*. Ed. D. W. Weiss. New York and London: Academic Press. p. 230.